# Human IgG3 with extended half-life does not improve Fc-gamma receptor-mediated cancer antibody therapies in mice

**DOI:** 10.1371/journal.pone.0177736

**Published:** 2017-05-19

**Authors:** Rens Braster, Simran Grewal, Remco Visser, Helga K. Einarsdottir, Marjolein van Egmond, Gestur Vidarsson, Marijn Bögels

**Affiliations:** 1 Department of Molecular Cell Biology and Immunology, VU University Medical Centre, Amsterdam, The Netherlands; 2 Department of Surgery, VU University Medical Centre, Amsterdam, The Netherlands; 3 Department of Experimental Immunohematology, Sanquin Research, and Landsteiner Laboratory, Academic Medical Centre, University of Amsterdam, Amsterdam, The Netherlands; Duke University School of Medicine, UNITED STATES

## Abstract

**Background:**

Current anti-cancer therapeutic antibodies that are used in the clinic are predominantly humanized or fully human immunoglobulin G1 (IgG1). These antibodies bind with high affinity to the target antigen and are efficient in activating the immune system via IgG Fc receptors and/or complement. In addition to IgG1, three more isotypes are present in humans, of which IgG3 has been found to be superior compared to human IgG1 in inducing antibody dependent cell cytotoxicity (ADCC), phagocytosis or activation of complement in some models. Nonetheless, no therapeutic human IgG3 mAbs have been developed due to the short *in vivo* half-life of most known IgG3 allotypes. In this manuscript, we compared the efficacy of V-gene matched IgG1 and IgG3 anti-tumour mAb (TA99) in mice, using natural variants of human IgG3 with short- or long half-life, differing only at position 435 with an arginine or histidine, respectively.

**Results:**

*In vitro* human IgG1 and IgG3 did not show any differences in opsonisation ability of B16F10-gp75 mouse melanoma cells. IgG1, however, was superior in inducing phagocytosis of tumour cells by mouse macrophages. Similarly, in a mouse peritoneal metastasis model we did not detect an improved effect of IgG3 in preventing tumour outgrowth. Moreover, replacing the arginine at position 435 for a histidine in IgG3 to enhance half-life did not result in better suppression of tumour outgrowth compared to wild type IgG3 when injected prior to tumour cell injection.

**Conclusion:**

In conclusion, human IgG3 does not have improved therapeutic efficacy compared to human IgG1 in a mouse tumour model.

## Introduction

The development of new and better monoclonal antibodies (mAb) to use as therapy to treat cancer—in addition to chemo- and/ or radiotherapy—has increased dramatically in the last decade [1]. mAbs can be designed to specifically interact with tumour-associated antigens, and initiate a wide range of effector mechanisms, which can potentially result in regression of the tumour. Several anti-tumour mAbs have now been approved for cancer therapy by the American food and drug administration (FDA), and the number of potential new targets is increasing rapidly. Successful mAb that are currently used in the clinic are anti-CD20 mAbs, which are widely employed in the treatment of several B-cell malignancies and significantly improve patient prognosis [2]. Treatment with the anti-CD38 mAb Daratumumab was shown to improve clinical outcome of patients with multiple myeloma [3]. Additionally, anti-HER-2/neu and anti- epidermal growth factor receptor mAbs are increasingly used to treat several malignancies such as mammary carcinoma, colorectal cancer or head and neck cancer respectively. However, despite some clinical successes, a substantial proportion of cancer patients fail to achieve complete remission or experience relapse after receiving mAb therapy. Improvement of antibody immunotherapeutic approaches is therefore warranted.

Therapeutic mAbs can trigger a multitude of functions to eliminate tumour cells. These include direct effects, such as inducing growth arrest of tumour cells by blocking the binding of growth factors to their receptor, or initiation of apoptosis [1,4,5]. In addition, after binding to tumour cells they can activate the complement system to destroy the target cell, which is referred to as complement dependent cytotoxicity (CDC). Through their Fc part, mAbs of the immunoglobulin G (IgG) isotype can furthermore bind to IgG Fc receptors (Fcγ receptors) that are expressed on immune effector cells. This can lead to killing of tumour cells via a process referred to as antibody dependent cellular cytotoxicity (ADCC) or through antibody dependent cellular phagocytosis (ADCP).

*In vivo*, Fcγ receptor mediated mechanisms were shown to play a prominent role in antibody-mediated (TA99) therapies against the syngeneic B16 melanoma model [6]. In this model, anti- tumour mAb treatment of Fc receptor γ chain- deficient mice, which lack the activating receptors FcγRI, FcγRIII and FcγRIV, did not protect against the development of lung metastases, whereas control wild type mice had less tumour development after mAb therapy [6]. We previously demonstrated that treatment of mice with murine IgG2a (which shares functional homology with human IgG1) successfully prevented the development of liver metastases. Clinical efficacy depended on the presence of FcγRI and FcγRIV [7]. Additionally, liver macrophages (Kupffer cells) were shown to be indispensable for mAb-induced eradication of tumour cells, which was due to effective ADCP [8,9].

Currently, most clinical mAbs are of the IgG1 isotype, due to favourable characteristics such as the ability to induce CDC, ADCC and ADCP, combined with its long half-life. Interestingly, human IgG3 has the highest affinity for Fcγ receptors and activates complement even more potently than IgG1 [10,11]. Hence, IgG3 has been found to mediate even stronger effector functions than IgG1 *in vitro* and in mice [12–14]. As such, it is in theory the best ligand for all Fcγ receptors expressed on macrophages and other immune cells [15]. IgG3 has however a short half-life *in vivo*, making it potentially less effective for mAb therapies [16]. It was demonstrated that human IgG1 antibodies, which are the most abundant IgG isotype in humans, inhibit FcRn-mediated transport and rescue of human IgG3, subsequently leading to lysosomal degradation of IgG3 [12,17]. Due to the shorter half-life and earlier assumptions that the longer hinge region of IgG3 would lead to enhanced proteolytic degradation no IgG3 therapeutic antibodies are under development so far [18,19]. However, as it was demonstrated that IgG3 induces superior phagocytosis of e.g. opsonised (infected) red blood cells or micro-organisms by mononuclear cells, IgG3 may represent an ideal candidate for antibody therapy to prevent the development of liver metastases [20,21]. We therefore developed recombinant V-gene-matched human IgG1 and IgG3 mAbs against murine B16 tumour cells, and evaluated their potential in prevention of tumour development. Additionally, we introduced an amino acid change in the Fc domain of IgG3 at position 435 that represents a natural variation also found in humans, which is essential for FcRn interactions [12,22]. All subclasses and IgG-species from other mammals have been found to have an histidine (H) at this position which endows IgG antibodies a pH-dependent binding to FcRn, which is crucial for their FcRn-dependent recycling properties [12,23]. IgG3 has classically been defined as a subclass with an arginine (R) at this position, which we recently found to be the cause of the short have-life of IgG3 due to competition with the other subclasses for FcRn-mediated transport [12]. However, due to an allotypic variation at this position in IgG3, some individuals actually do have histidine at this position. Unlike R435-expressing individuals, IgG3 in H435-expressing individuals has a half-life comparable to IgG1. In addition, placental transport, which is also mediated by FcRn, of IgG3 in humans is also comparable to IgG1 in H435-IgG3 expressing individuals, strongly supporting that this amino acid variation is the underlying factor for the reported short half-life for IgG3 [24].

As we and others have found IgG3 to have strong effector functions through both Fcγ receptor [11,12] and complement [11,13,25], we now tested if H435-IgG3 has superior effector functions as therapeutic antibody (TA99) in the B16 melanoma model.

## Materials and methods

### Animals

Male C57Bl/6 mice, weighing 20–25 g, were obtained from Envigo, Boxmeer, The Netherlands. Animals were kept under standard laboratory conditions and had access to food and water ad libitum. The Committee for Animal Research of the VUmc approved all experiments, according to the European and national guidelines. Animals were daily monitored for signs of discomfort including: ≥15% weight loss; activity and personal hygiene. No change in behaviour was observed during the experiments. Tumour growth could not be followed over time due to the location, however, the model was setup to score single tumours within the peritoneal cavity.

### Cell culture

#### Tumour cells

Truncated GP75 (Tyrp-1, tyrosinase related protein 1) was transfected in mouse melanoma B16F10 (B16F10-gp75) cells as described in [9] and cultured in RPMI-1640 (Invitrogen, Paisley, UK), supplemented with 10% heat inactivated foetal calf serum (FCS), penicillin (100 U/ml), streptomycin (100 μg/ml) and L-glutamine (200 μM) (further referred to as complete RPMI). Cell suspensions were prepared by enzymatic detachment using trypsin-EDTA solution (Invitrogen). Viability was assessed by trypan blue exclusion and always exceeded 95%.

#### L929 cell conditioned medium (LCM)

Mouse L929 cells secrete macrophage colony-stimulating factor (M-CSF) and were used to produce L929 cell conditioned medium (LCM) to differentiate macrophages. L929 cells were grown to confluency, after which medium was changed with fresh complete RPMI. Cells were grown for 7 days, after which LCM was harvested, centrifuged at 4750xg for 10 minutes, filtered through 0.2 μm filters and stored at -20°C till further use.

#### Murine bone marrow macrophages

Bone marrow was harvested from freshly isolated femur, tibia and humerus from wild type C57BL/6. After removal of connective tissues and muscles, bone marrow was flushed and single cell suspensions were made by passing bone marrow through a sterile 70 μm filter (BD Falcon, Bedford, MA). Macrophages were differentiated by incubating bone marrow cells for 7 days with complete DMEM, supplemented with 15% LCM (hereafter referred to as macrophage medium). Macrophages were harvested after 15 minute incubation with trypsin-EDTA and subsequent scraping using a cell scraper. Macrophages were seeded in 24 well plates (4x10^5^/ well) for *in vitro* cytotoxicity assays.

### Generation of human IgG1 and IgG3 anti-GP75 (TA99)

The variable regions of the heavy and light chains (VL, VH) were cloned from a hybridoma, which produces murine IgG2a mAb against the murine gp75 antigen. Variable regions were expressed as chimeric human IgG in a similar manner as previously described [11]. RNA was isolated with the RNeasy Mini Kit, (Qiagen, CA), and VH and VL genes were amplified as described in [26], with CH and CL specific primers. The product was then ligated into pGEM-T (Invitrogen, CA) and sequenced by ABI 373 Stretch automated sequencing (Applied Biosystems, Foster City, CA). Codon optimized V-genes, including 5’HindIII, and 3’EcoRI restriction sites, Kozak sequence and HAVT20-leader [27] were then designed and ordered from MR Gene (now Geneart/Invitrogen), along with codon optimized human κ, γ1, γ3 constant regions for the variable light and heavy chains, respectively. H/R mutations were introduced at position 435 as described by Stapleton et al [12]. HindIII/EcoRI fragments for the codon optimized light chain was ligated into pEE14.4 (Lonza), the HindIII/EcoRI heavy chain into pEE6.4 (Lonza). The correct assembly of the final construct was verified by sequencing, and it produced in the FreeStyle 293 expression system (Invitrogen) according to the manufacturer’s instructions. Antibodies were purified on a protein A (H435 IgG) or protein G (R435 IgG) HiTrap HP column (GE Life Sciences) and dialyzed against PBS overnight. As a result, we obtained IgG1 with a serology known as G1m(f), IMGT code IGHG1*03, and IgG3 with a serology known as G3m(g*), IMGT code IGHG3*16.

### IgG quantification

IgG was quantified by sandwich ELISA using subclass specific mouse monoclonal antibodies (IgG1:MH161-1; IgG3:MH163-1, Sanquin) for capture. Mouse-anti-IgG-HRP (Southern Biotech, Birmingham, AL) was used for detection.

### Flow cytometry

Mouse B16F10 or B16F10-gp75 cell lines were incubated with different concentrations of primary mouse or human anti-gp75 mAb or human IgG1 anti-HEPC (isotype control) or mouse IgG2a anti-rat MG4 (isotype control) antibodies for 45 minutes on ice. After washing, primary antibody was detected by incubation with PE-conjugated goat-anti-human IgG mAb (1:50) or rabbit-anti-mouse IgG mAb. Cells were analysed with flow cytometry (FacsCalibur, BD, San Jose, CA).

### Fluorescent labelling

For *in vitro* cytotoxicity assays mouse tumour cells were harvested and incubated (1–10x10^6^ cells/ml) in complete DMEM supplemented with 2.5 μg/ml 1,1’-dioctadecyl-3,3,3’,3’-tetramethylindocarbocyanine perchlorate (DiI, Sigma-Aldrich, St. Louis, MO) for 30 minutes at 37°C, and subsequently washed three times with complete DMEM. Macrophages were incubated (1–10x10^6^ cells/ml) in complete Macrophage medium, supplemented with 2.5 μg/ml 3,3’-dioctadecyloxacarbocyanine perchlorate (DiO, Molecular Probes Inc, Paisley, UK) for 30 minutes at 37°C and subsequently washed three times with complete macrophage medium.

### In vitro cytotoxicity assays

Cytotoxicity assays were performed by co-culturing DiI-labelled mouse B16F10 or B16F10-gp75 tumour cells and DiO-labelled macrophages in an effector to target (E:T) ratio of 15:1 in the presence of different concentrations of mouse anti-gp75 mIgG2a or human hIgG1, hIgG3, hIgG1-H435R or hIgG3-R435H. Human anti-HEPC IgG1 or mouse anti-Rat MG4 IgG2a antibodies served as isotype controls. Percentages of remaining tumour cells and macrophages that had taken up tumour cells (double-positive) were determined by flow cytometry after 24 hours of co-culture. Percentages of tumour cells after culture with isotype antibodies were set at 100%. Double-positive macrophages were depicted relative to the co-cultures with isotype antibodies (set to 1), as described previously [4].

### PMN isolation

Human PMNs were isolated from peripheral blood that was obtained from healthy donors. All donors signed an informed consent, according to guidelines of the Medical Ethical Committee of the VUmc. Blood was separated by Lymphoprep (Axis-Shield, Oslo, Norway) density gradient centrifugation. PMNs were obtained by lysing erythrocytes in ammonium chloride buffer (155 mM NH4Cl, 10 mM KHCO3 and 0.1 mM EDTA). Subsequently, PMNs were washed with PBS (B.Braun, Melsungen, Germany) and resuspended in complete medium.

### Antibody dependent cellular cytotoxicity (ADCC)

B16F10-gp75 tumour cells were seeded in 96 wells plates (8.000 cells/well). PMNs were added after 24 hours (effector to target (E:T) ratio 80:1) in the presence or absence of anti-gp75 antibodies of different isotypes. After 4hr of co-culture plates were carefully washed and a 3-hours cell titre blue assay (Promega, Leiden, the Netherlands) was performed according to manufactures protocol.

### Animal model

The used mouse peritoneal tumour model has been adapted from a peritoneal tumour model in rats, which we previously described [28]. Mice were intraperitoneally (i.p.) injected with antibodies diluted in PBS (50μg/300μl) 4 days prior to injection of the tumour cells. B16F10-gp75 cells were harvested at the start of the experiment, washed and diluted in PBS to a concentration of 50.000 cells/300μl and i.p. injected. Mice were sacrificed 14 days post injection with CO_2_ and peritoneal tumour load was scored according to the scoring system as previously described [28]. Briefly, the diameter of each tumour node was measured with a calliper. Tumour load was defined as the sum of the diameters of all tumour nodules per mouse in mm.

### Statistical analysis

Data was analysed with Bonferroni-Post Hoc tests, preceded by two-way ANOVA tests for comparison of multiple groups. Significance was accepted at p < 0.05.

## Results and discussion

### Antibody modification does not affect the binding capacity

**Fig 1:**
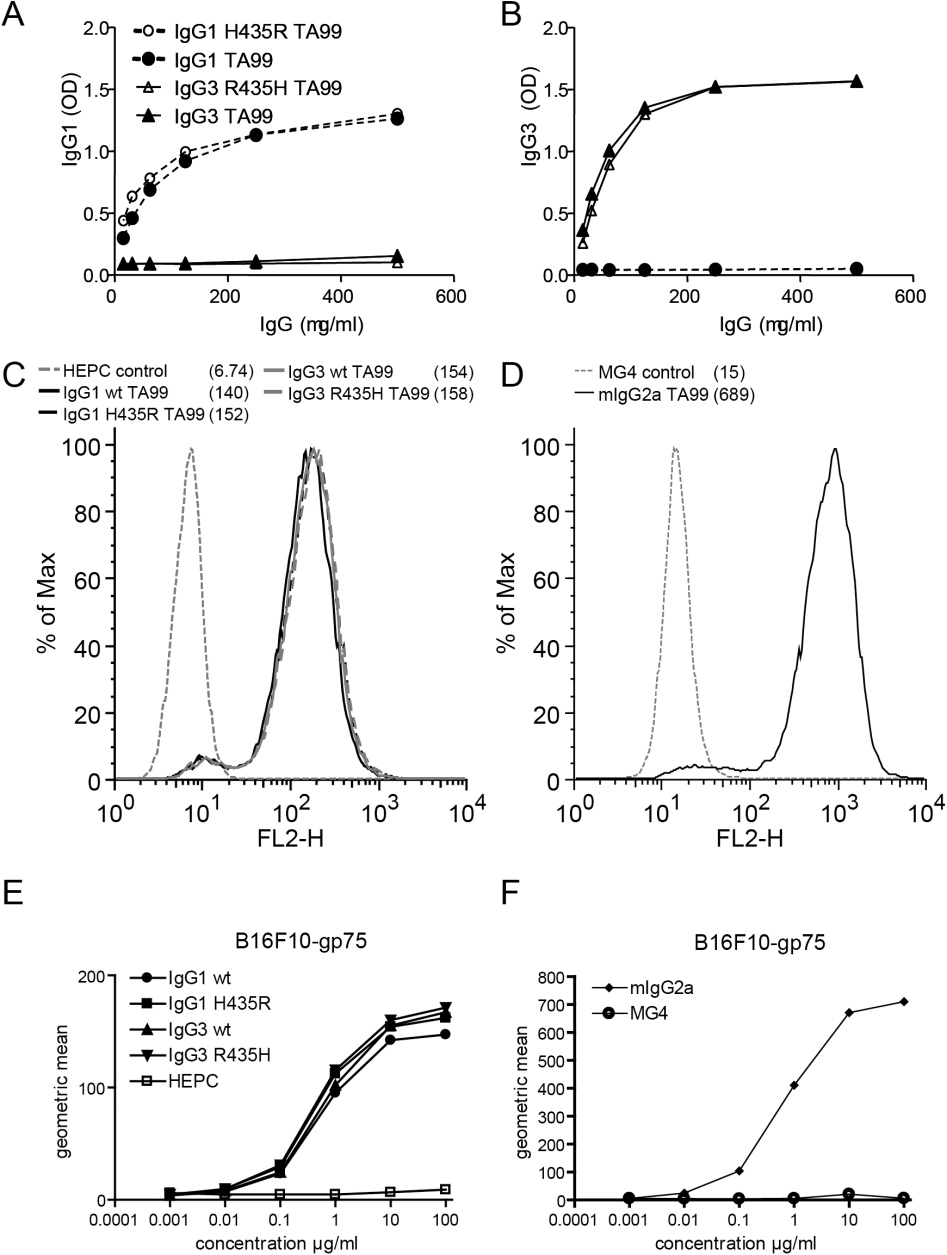
Generation of human IgG1 and IgG3 TA99 mAb with histidine-arginine rearrangements in Fc domains at amino acid position 435. Human IgG1 was mutated to contain an arginine at position 435 (IgG1 H435R), whereas the arginine at position 435 in human IgG3 was changed to histidine (IgG3 R435H). (**A**) Specific anti-human IgG1 or (**B**) anti-human IgG3 ELISA confirmed the correct isotype of mAbs. Staining of B16F10-gp75 with (**C**) different human TA99 mAb and (**D**) mouse TA99 IgG2a confirmed binding to surface gp75 and equal binding efficiency to gp75 of all human TA99 mAb. Concentration curves of human IgG1 and IgG3 mAb (**E**) and mouse IgG2a TA99 (**F**) on B16F10-gp75. Of note, scales of human (**E**) and mouse (**F**) antibodies are different.

One potential drawback of using IgG3 isotype mAbs as therapeutic antibodies is the fact that human IgG1 (hIgG1) inhibits the recycling of hIgG3 via the neonatal FcRn, leading to a short half-live of IgG3 [12]. Although FcRn binds both Fc domains of hIgG1 and hIgG3 with comparable affinity, the difference at position 435 in the Fc fragment of hIgG3 (histidine in IgG1 versus arginine in hIgG3) accounts for a decreased competitiveness of hIgG3 for pH-dependent FcRn binding and release, leading to increased hIgG3 lysosomal degradation [12,23]. To overcome the inferior half-life of wild-type hIgG3 we constructed a specific IgG3 containing a histidine at position 435 in its Fc domain, as it has been demonstrated that this leads to enhanced rescue through FcRn and subsequently to increased half-life *in vivo* in FVB mice [12]. The neonatal Fc receptor is a highly-conserved molecule. A sequence comparison of this receptor between FVB, C57Bl/6 and Balb/C demonstrated that C57Bl/6 and FVB have identical sequences in all aspects when it comes to IgG-contact and all other functional residues (data not shown, accession numbers AAA16904, NP_034319, BAA07110 and ref. [29]). Only one amino acid difference was observed that is, however, well outside of the functional- or IgG contact region, and as such unlikely to influence the interaction of IgG with FcRn in C57Bl/6 mice [29]. In both the humanized hIgG1 TA99 as the hIgG3 TA99 mutations (histidine to arginine and vice versa respectively) were introduced at position 435 generating hIgG1-H435R TA99 and hIgG3-R435H TA99. Specific anti-human IgG1 or anti-human IgG3 ELISA confirmed the correct isotype of the mAbs (Fig 1A and 1B). Additionally, we confirmed with SDS-PAGE that the structural integrity was not affected due to this amino acid modification (S1 Fig).

Next, the binding and functionality of human IgG1 and IgG3 TA99 variants was investigated. TA99 is directed against the 75-kD product of the *brown* gene, known as TRP-1 or glycoprotein gp75. This is highly conserved between mouse and human and expressed in melanocytic cells, like the mouse B16F10 melanoma cell line [30]. Gp75 is however exported efficiently towards intracellular melanosomes and almost no gp75 protein reaches the cell surface *in vitro* [7,30–32]. As such, mouse macrophages were not able to phagocytose B16F10 cells in the presence of TA99 *in vitro* (data not shown). It is possible that gp75 expression on B16F10 is up regulated *in vivo*, accounting for the *in vivo* efficacy seen with the anti-GP75 mIgG2a antibody [33]. This is supported by experiments performed with fresh B16F10 cells isolated from lung metastases, which had enhanced surface expression of gp75, and successful ADCC of these cells was induced through mIgG2a TA99 mAb *in vitro* [34]. Therefore, it is conceivable that *in vivo* TA99 mAbs are able to efficiently bind B16F10, and induce killing via high affinity interaction with murine Fcγ receptors. For *in vitro* studies, we therefore generated a cell line that stably expresses a truncated gp75 protein on the membrane by removing the dileucine intracellular retention signal motif which is critical for internalization (referred to as B16F10-gp75) [9,32]. For consistency, all experiments (both *in vitro* and *in vivo*) were performed with these cells. TA99 hIgG1-H435R or hIgG3-R435H isoforms recognized membrane expressed gp75 on B16F10-gp75 cells similarly (Fig 1C and 1D). In addition, titration of humanized IgG antibodies revealed similar binding curves, compared to each other (Fig 1E) and compared to the original TA99 mouse IgG2a antibody (Fig 1F), indicating similar affinities.

### IgG1 is more efficient in inducing tumour cell kill in vitro with mouse macrophages

**Fig 2:**
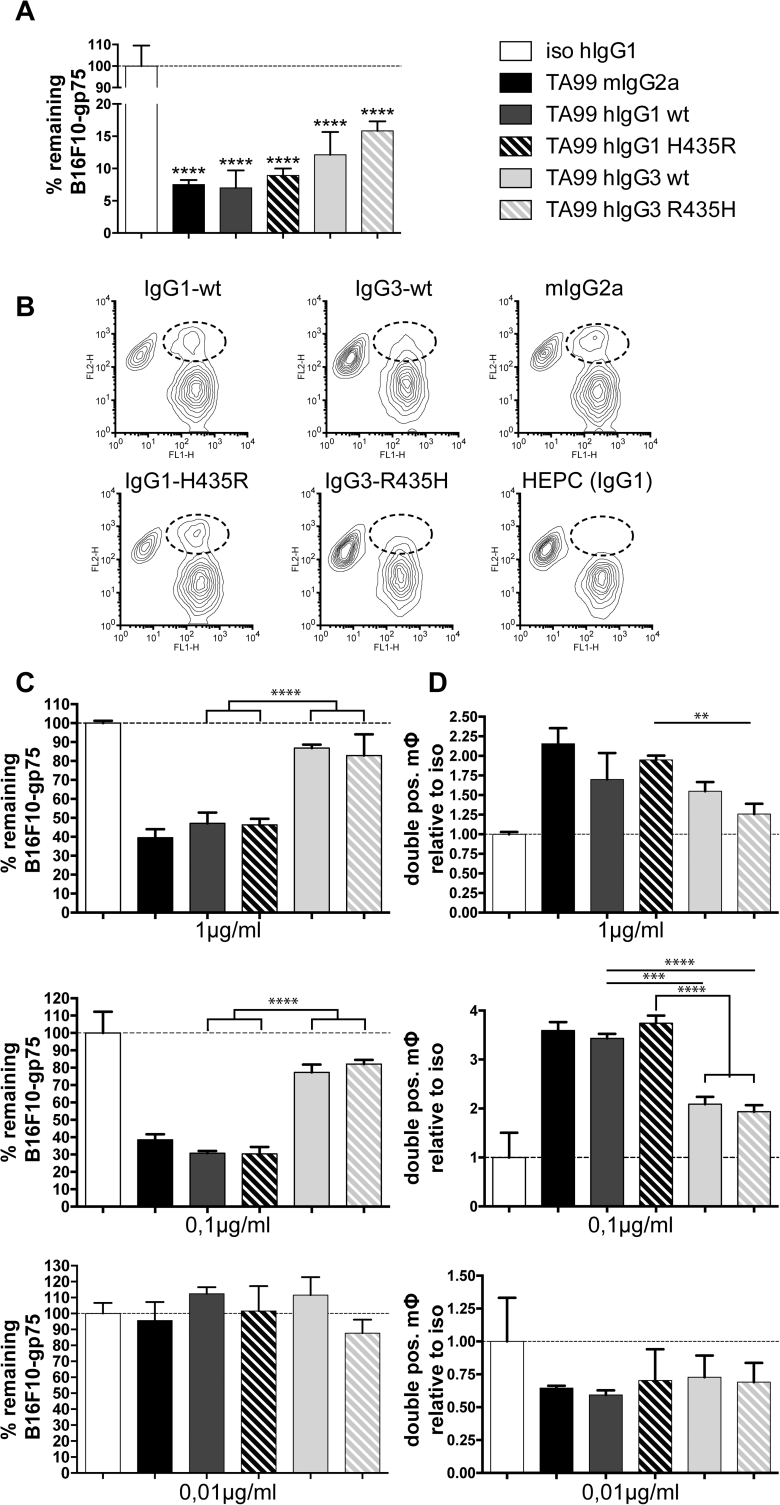
Cytotoxicity assays using murine macrophages and B16F10-gp75 tumour cells. (**A**) Remaining B16F10-gp75 cells after a 24 hour incubation with macrophages and 2 μg/ml TA99 mAbs (different isotypes). (**B**) FACS analysis of co-cultures of DiO labelled murine macrophages (FL1) and DiI labelled B16F10-gp75 (FL2) tumour cells after 24 hours of treatment with 1 μg/ml mouse IgG2a or human IgG1 or IgG3 TA99 mAb. Macrophages, which have phagocytosed B16F10-gp75 tumour cells are encircled in FACS plots. (**C**) Percentage of remaining viable tumour cells and (**D**) increase in number of macrophages, which have phagocytosed B16F10-gp75 tumour cells after treatment of co-cultures with different concentrations of mAb. Percentages of tumour cells after culture with isotype antibodies were set at 100%. Double-positive macrophages were depicted relative to the co-cultures with isotype antibodies (set to 1), as described previously [4]. Mouse MG4 or human HEPC mAb were used as isotypes controls, which were set to 100%. *P<0.05, **P<0.01, ***p<0.001, ****p<0.0001.

The efficiency of TA99 antibodies *in vitro* was tested by co-culturing mouse macrophages with the newly generated B16F10-gp75 cells in the presence of antibodies. At an antibody concentration of 2 μg/ml, hIgG1 and hIgG3 were equally efficient in inducing macrophage mediated tumour killing, with IgG1 mediating perhaps a slightly improved, but not significantly different, killing (Fig 2A).

Other studies demonstrated enhanced phagocytosis of red blood cells in the presence of hIgG3 [20,21,35–38]. Moreover, opsonisation of *S*. *Typhimurium* with hIgG3 resulted in enhanced bacterial uptake by the monocyte cell line THP-1, which was dependent on FcγRI expression [39]. We therefore tested whether TA99 hIgG3 was more efficient in B16F10-gp75 tumour cell killing and phagocytosis at lower mAb concentrations.

Lower concentrations of 0.1 μg/ml hIgG1 or mIgG2a TA99 still induced B16F10-gp75 killing by mouse macrophages through phagocytosis (Fig 2B–2D middle panel). Both hIgG1 and mIgG2a were equally able to induce tumour cell phagocytosis by macrophages (Fig 2C and 2D). By contrast, B16F10-gp75 killing was significantly reduced in the presence of human IgG3 TA99 mAb concentrations of 1 μg/ml or lower (Fig 2C and 2D). *In vitro*, histidine or arginine rearrangements at amino acid position 435 in either hIgG1 or hIgG3 TA99 did not influence B16F10-gp75 killing (Fig 2C and 2D).

Thus, B16F10-gp75 tumour cells were efficiently killed by macrophages in the presence of either IgG1 TA99 or IgG3 TA99. We demonstrated with intravital microscopy that elimination of circulating B16F10-gp75 cells was mainly mediated via ADCP by liver macrophages (Kupffer cells) [9]. Furthermore, live cell imaging and ImageStream analysis of *in vitro* co-cultures identified ADCP as a major killing mechanism of macrophages [4]. Formally we cannot exclude binding of tumour cells to the outside of the macrophages without killing. However, in our previous analyses we demonstrated that the vast majority of double positive macrophages had taken up tumour cells and only minimal binding outside macrophages was observed [4]. The percentages of double-positive macrophages (doubled when IgG1 was added compared to isotype control: Fig 2D) was relatively low to account for ~60% reduction in tumour cells. It was demonstrated that not all macrophages phagocytose tumour cells, whereas other macrophages take up multiple tumour cells, which may explain this discrepancy [40]. Alternatively, it was shown that macrophages can kill via ADCC, as well [41]. Synapse formation between F4/80 expressing cells (macrophages) and tumour cells, which is indicative of ADCC, was observed *in vivo* after treatment with therapeutic antibodies [42]. As such, the disparity between the reduction of tumour cells and the doubling of double-positive macrophages may be partly due to ADCC.

We additionally performed ADCC experiments with polymorphonuclear cells (PMNs), as they have been proposed as potential effector cells. Because mice only have a low number of circulating PMNs (<10%), we used isolated human PMNs. It was previously shown that IgA antibodies induce higher tumour cell killing by PMNs, compared to IgG [43,44]. We therefore included IgA-TA99 in the experiment. We observed some killing (20%) of B16F10-gp75 mouse melanoma cells in the presence of IgA-TA99. However, neither IgG1 TA99 nor IgG3 TA99 induced any killing by PMNs (S2A Fig). [43,44]. Additionally, as measure for neutrophil activation, we determined lactoferrin (degranulation marker) in the supernatant (S2B Fig). No differences were observed when PMNs were incubated with B16F10-gp75 tumour cells in the presence of specific TA99 mAbs or irrelevant isotype antibodies, supporting that PMNs were not activated.

### Both human IgG1 and IgG3 inhibit formation of peritoneal tumours

**Fig 3:**
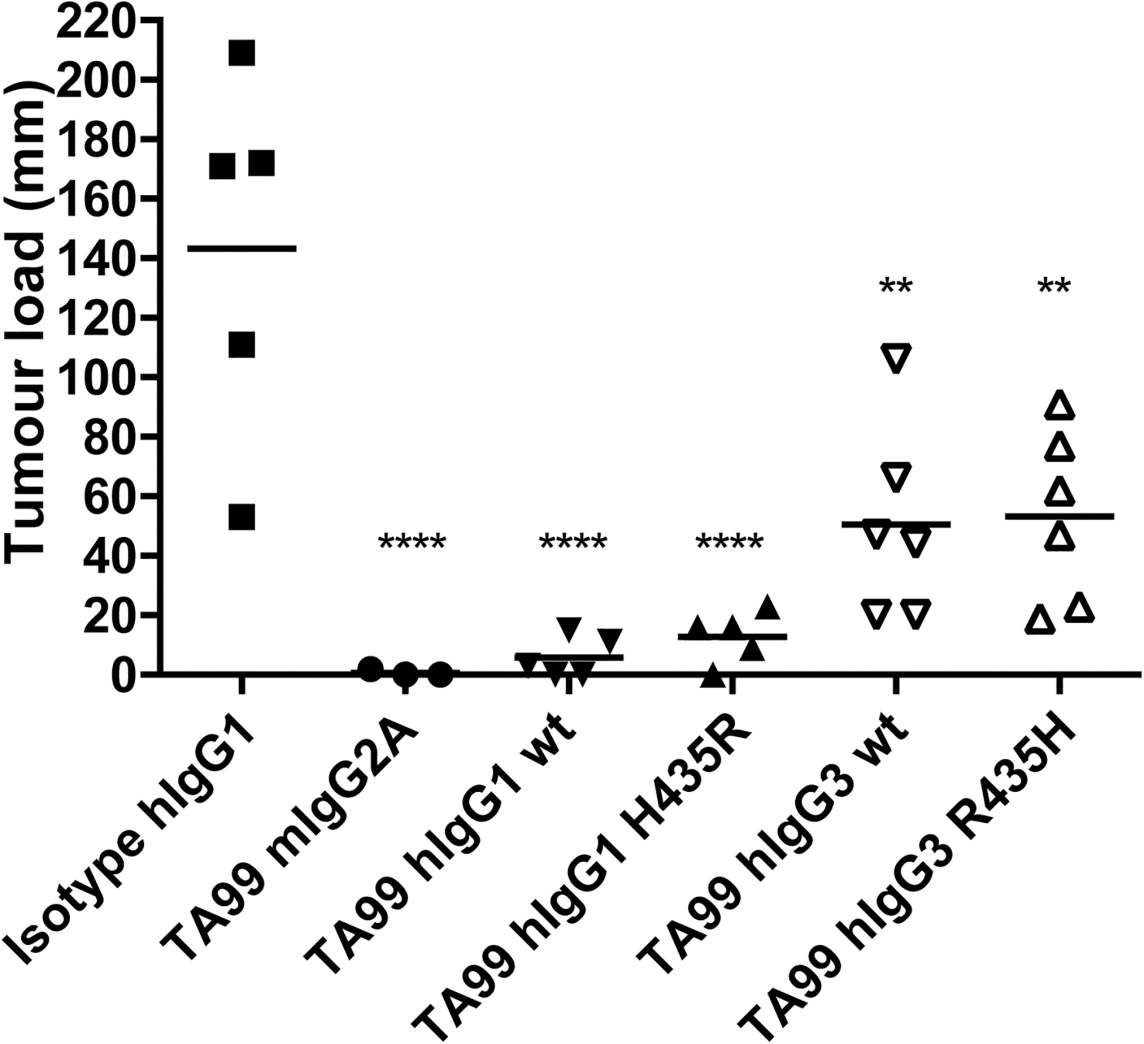
Prevention of B16F10-gp75 metastases development with different TA99 isotype antibodies. Mice received peritoneal injections with 50μg mAb/mouse 4 days prior to peritoneal injection of B16F10-gp75 tumour cells. Metastases outgrowth after treatment with TA99 mouse IgG2a or (modified) human IgG1, human IgG3 mAbs was determined 14 days post tumour cell injection. **P<0.01, ****p<0.0001.

To study if TA99 hIgG3 has an enhanced effect over TA99 hIgG1 and whether TA99 hIgG3-R435H overcomes the difference in half-life with IgG1, an intra-peritoneal (i.p.) metastasis model of B16F10-gp75 tumour cells was used similar to what we previously described [28]. The antibody dose was determined in a titration experiment and a dose of hIgG1 TA99 with suboptimal effect was selected, as this would allow demonstration of the hypothesized improved effect of hIgG3 TA99 (data not shown). As we anticipated that hIgG3 TA99 had a shorter half-life compared to hIgG1, we selected day -4 for antibody therapy, as at this time point sufficient hIgG1 TA99 or TA99 hIgG3-R435H (but less so for hIgG3 TA99) would still be present at the day of tumour cell inoculation [12]. To prevent the development of mouse anti-human antibodies (MaHa), which would interfere with the model, we did not go beyond 4 days prior to injection of B16F10-gp75 cells. Tumour growth was assessed 14 days post tumour cell injection. Mice treated with either mIgG2a or hIgG1 had almost no metastasis outgrowth (Fig 3). TA99 hIgG3, however, was less effective in preventing tumour outgrowth. Moreover, due to the introduced mutation in the TA99 hIgG1 recycling was expected to be reduced resulting in a decreased half-life. As a result, we hypothesised that this antibody would be less effective than the wildtype hIgG1. However, no difference between the wild type and the mutant was observed. Furthermore, mice treated with TA99 hIgG3 R435H were not better protected against tumour outgrowth compared to TA99 hIgG3 wildtype.

These results were surprising, as hIgG3 is superior in inducing complement subunit C1q binding compared to hIgG1 [19,45,46]. Subsequently, C1q can activate the classical complement route, leading to complement induced lysis of tumour cells. Additionally, immune responses can be induced by binding either directly to complement receptor 1 (CR1/CD35) on effector cells or via forming a complex with complement subunit 3, activating immune cells via complement receptor 3 (CR3/Mac-1/CD11b+CD18) [47]. CR1 is expressed by many different cells, including PMNs, monocytes, macrophages, and DCs but also B-cells and a subpopulation of CD4^+^ T-cells [48]. Proliferation of B-and T cells is regulated by interaction of CR1 with C1q immune complexes, whereas PMNs, monocyte/macrophages and DCs are able to phagocytose complement opsonized immune complexes such as tumour cells. Mac-1/CR3 is primarily expressed on monocytes/macrophages and neutrophils, binds C3bi—the cleaved form of C3—and contributes to ADCC. It was demonstrated that CD11b deficient mice that lack Mac-1 were less protected by treatment with mIgG2a TA99 mAb against B16F10 lung metastases development than wild-type mice [49]. Furthermore, although neutrophils were still able to penetrate malignant tissues and interacted with antibody opsonised tumour cells in Mac-1 -/- mice, they were unable to initiate ADCC towards tumour cells. Mac-1 on myeloid cells, including neutrophils therefore may play an important role in complement mediated tumour rejection. Because hIgG3 mAb were most effective in activating a respiratory burst in PMN, it is possible that the mechanism of action of hIgG3 in tumour rejection is dependent on both complement binding and PMN activation [11,50]. Mouse neutrophils however are not efficiently activated via IgG antibody. Therefore, mouse neutrophils are not likely to be effector cells in mAb-therapies tested in mouse models, which corresponds with the absence of *in vitro* killing of tumour cells by human PMNs.

Previously, we and others, demonstrated that mAb therapy of B16F10 liver metastases with mIgG2a TA99 (functional homologous to hIgG1) was independent of complement activation, but relies on Fcγ receptor and macrophage presence [7,51]. This may be due to expression of complement regulatory proteins, like CD55 and CD59 that can be expressed by tumour cells, and suppress CDC. It was shown that blocking these two molecules enhanced both CDC and ADCC [52].

Since treatment with IgG3 did not enhance therapeutic success, potential enhanced IgG3-induced CDC likely did not occur in the B16F10-gp75 model.

Nonetheless, in addition to ADCP and ADCC, various studies demonstrated that complement can play a role in mAb therapy of cancer by inducing CDC, and enhancing ADCC (reviewed in [53,54]). For instance, it was shown that polymorphisms in the C1QA gene correlated with clinical responses in patients with follicular lymphoma after anti-CD20 mAb therapy with rituximab, supporting the importance of CDC [55]. Several second-generation therapeutic antibodies, such as the anti-CD20 mAb ofatumumab induce CDC more potently compared to rituximab. It has furthermore been demonstrated that transferring parts of the hIgG3 Fc domain into hIgG1 anti-CD20 mAb enhanced both complement binding, CDC and ADCC against lymphoma cells [13,14,25], supporting that several effector functions of mAb therapy were improved.

Recently a study with chimeric anti-transferrin receptor 1 mAbs with an hIgG3 Fc domain demonstrated which amino acids are essential for both induction of ADCC and CDC [56]. When the leucines at position 234 and 235 were mutated into alanines (L234A, L235A) and the proline at position 331 into a serine (P331S) ADCC was significantly reduced. Mutating the P331S alone was sufficient to prevent CDC almost completely. Other studies demonstrated that IgG3 is superior in mediating complement activation and that the length of the hinge matters [57,58]. Additionally, correlations between the length of the hinge of IgG3 and activation of the complement at low antigenic- concentrations were made. This may enable hexamer-formation,—required for efficient C1q binding -, more efficiently at low epitope density [59,60]. This is supported by a recent report showing that CD20-specific IgG3 are indeed more efficient than IgG1 in mediating CDC against tumour targets–especially on those targets with low tumour antigen-expression such as chronic lymphocytic leukaemia (CLL). Thus, it is possible that IgG3 mAb therapy may improve tumour cell killing of CDC-sensitive malignancies.

A recent study has classified binding affinities of human IgG subclasses to mouse Fcγ receptors, and found human IgG3 to bind with superior affinity to all mouse Fcγ receptors except mouse FcγRI, to which IgG1 bound better [40]. Nevertheless, in our hands IgG1 was in most cases superior to that of IgG3 in mediating in *in vitro* and *in vivo* effector functions. A reason for this discrepancy has not yet been put forward, but may lay in the fact that increased binding of human IgG3 was also found for the inhibitory FcγRIIb, which may be expected to impede Fcγ receptor-mediated activities, without affecting complement-mediated functions.

In conclusion, humanized IgG3 did not show improved Fcγ receptor-dependent tumour cell killing with mouse effector cells *in vitro* and *in vivo*. Although literature suggests that IgG3 can have superior effector functions, this is likely mediated through activation of complement. Thus, although tumours that are susceptible for CDC may be killed more effectively via IgG3, Fc receptor-mediated functions will likely be less efficient. Overall, IgG3 mAbs may therefore be less suitable for antibody therapy of cancer.

## Supporting information

S1 Fig Structural integrity of hIgG1 and hIgG3 were not affected. Human IgG1 contains a histidine at position 435 to secure normal recycling via FcRn in vivo, whereas human IgG3 contains an arginine at this position, which hinders IgG3 recycling. Human IgG1 was mutated to contain an arginine at position 435 (IgG1 H435R), whereas the arginine at position 435 in human IgG3 was changed to histidine (IgG3 R435H). Non-reducing (left panel) and reducing (right panel) page-gel electrophoresis confirmed structural integrity of the different IgG1 and IgG3 mAbs.
(TIF)

S2 Fig Human PMNs do not eliminate tumour cells in the presence of hIgG1 or hIgG3. (**A**) Human PMNs were co-cultured for four hours with B16F10-gp75 tumour cells in the presence of anti-gp-75 antibodies of different isotypes, after which the number of viable tumour cells was determined. Viability of tumour cells is relative to the no antibody control. (**B**) Lactoferrin release in supernatants of ADCC experiments with neutrophils was determined with ELISA (according to Aleyd et al. J. Immunol. 197:4552–59, 2016). No major differences were observed in lactoferrin release in response to tumour cells in the presence of specific TA99 mAbs or non-specific isotype control antibodies.
(TIF)

S1 Raw data Raw data for displayed figures in manuscript. (PDF)

## Abbreviations

ADCC: Antibody dependent cell cytotoxicity
ADCP: Antibody dependent cell phagocytosis
CDC: complement dependent cytotoxicity
E:T: Effector to target
FcRn: Neonatal Fc receptor
Fcγ receptor: IgG Fc receptor
GP75: glycoprotein75
Tyrp-1: tyrosinase related protein 1
i.p.: Intra peritoneal
IgG: Immunoglobulin G
LCM: L929 cell conditioned medium
M-CSF: Macrophage colony stimulating factor
mAb: monoclonal antibody
NK cell: Natural Killer cell
PMN: polymorphonuclear cells
