# Supplementary figures and images for: Human IgG3 with extended half-life does not improve Fc-gamma receptor-mediated cancer antibody therapies in mice
Source: PLoS One. 2017 May 19;12(5):e0177736. doi: 10.1371/journal.pone.0177736 (PMC5438146; doi:10.1371/journal.pone.0177736)

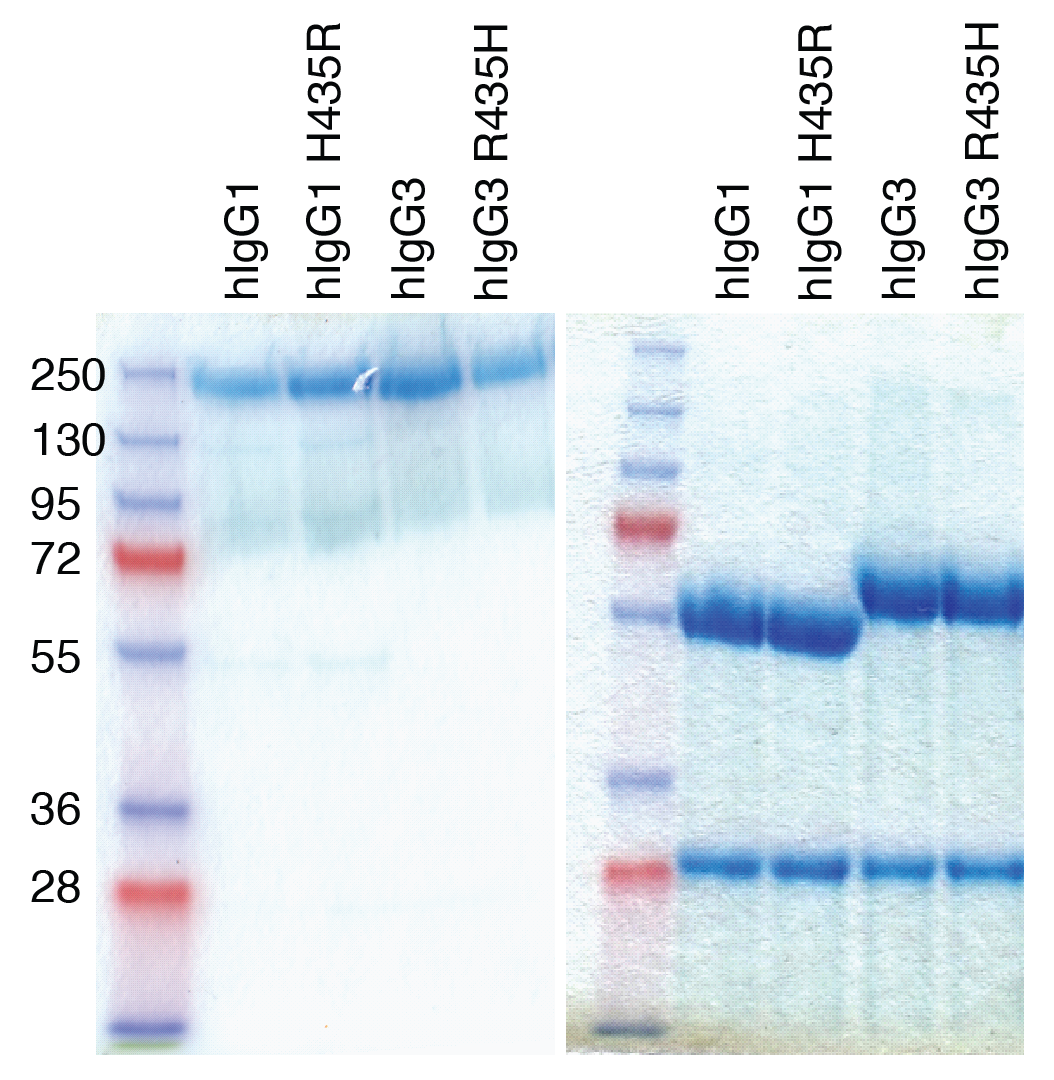

Supplement: S1 Fig — Human IgG1 contains a histidine at position 435 to secure normal recycling via FcRn in vivo, whereas human IgG3 contains an arginine at this position, which hinders IgG3 recycling. Human IgG1 was mutated to contain an arginine at position 435 (IgG1 H435R), whereas the arginine at position 435 in human IgG3 was changed to histidine (IgG3 R435H). Non-reducing (left panel) and reducing (right panel) page-gel electrophoresis confirmed structural integrity of the different IgG1 and IgG3 mAbs. (TIF) [file pone.0177736.s001.tif]

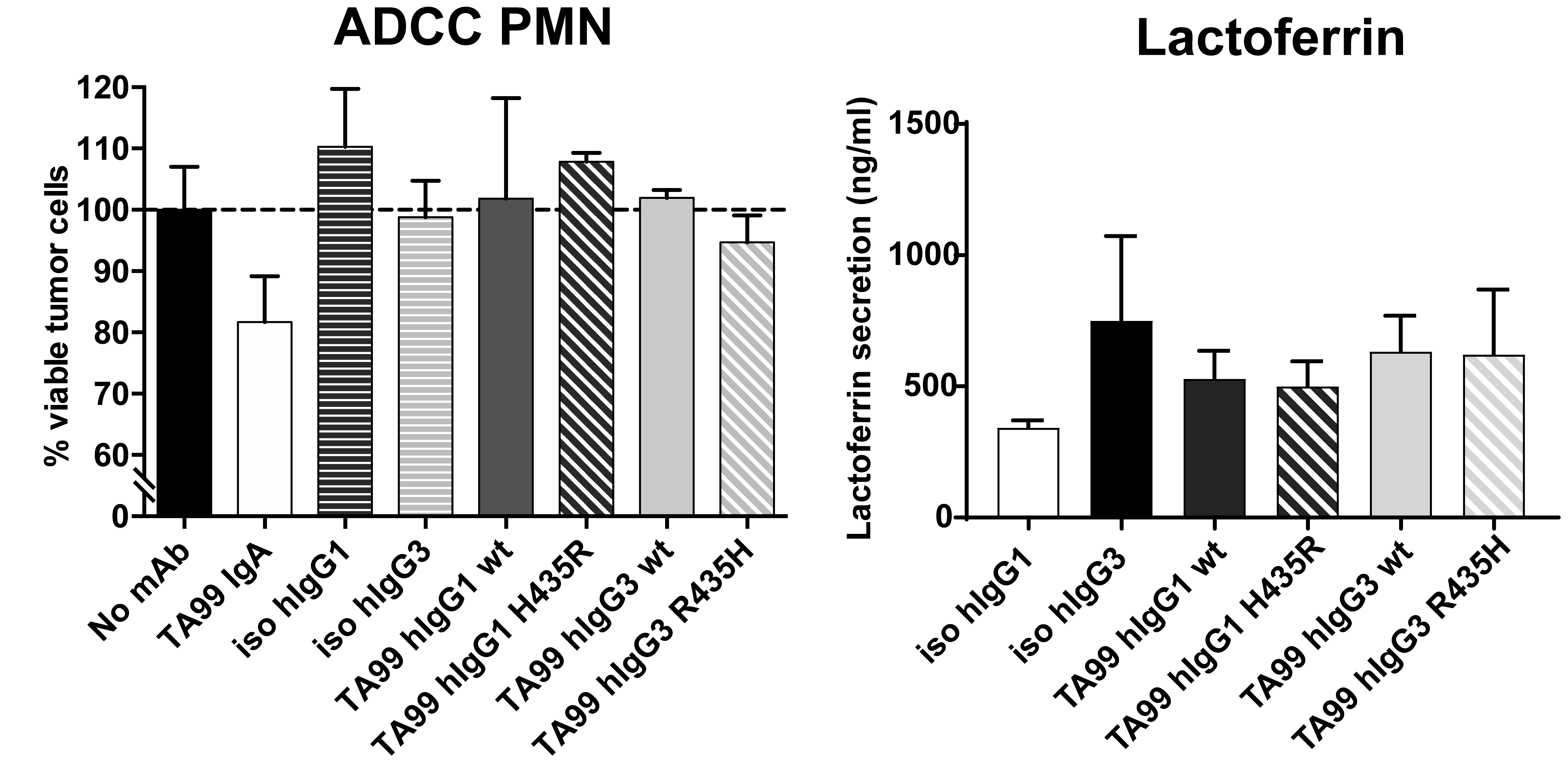

Supplement: S2 Fig — (A) Human PMNs were co-cultured for four hours with B16F10-gp75 tumour cells in the presence of anti-gp-75 antibodies of different isotypes, after which the number of viable tumour cells was determined. Viability of tumour cells is relative to the no antibody control. (B) Lactoferrin release in supernatants of ADCC experiments with neutrophils was determined with ELISA (according to Aleyd et al. J. Immunol. 197:4552–59, 2016). No major differences were observed in lactoferrin release in response to tumour cells in the presence of specific TA99 mAbs or non-specific isotype control antibodies. (TIF) [file pone.0177736.s002.tif]
