# Supplementary material for: Human IgG3 with extended half-life does not improve Fc-gamma receptor-mediated cancer antibody therapies in mice
Source: PLoS One. 2017 May 19;12(5):e0177736. doi: 10.1371/journal.pone.0177736 (PMC5438146; doi:10.1371/journal.pone.0177736)
Supplement: S1 Raw data — (PDF) [file pone.0177736.s003.pdf]

S3 Raw data

Raw data for figure 1

Figure 1A

	Optical density			
gG1 (ng/ml	1 H435R T/	G1 WT TA(	3 R435H T/	G3 WT TA99
500	1.30255	1.2636	0.1026	0.15375
250	1.13465	1.13055	0.09375	0.1111
125	0.99555	0.91915	0.08905	0.0949
62.5	0.78325	0.68895	0.0942	0.09355
31.25	0.63555	0.4596	0.08865	0.0929
15.625	0.43975	0.299	0.0948	0.09235

Figure 1B

	Optical density			
IgG3	1 H435R T/	G1 WT TA(	3 R435H T/	G3 WT TA99
500	0.05165	0.0525	1.566	1.56775
250	0.04575	0.04515	1.519	1.52305
125	0.04505	0.0423	1.3019	1.3527
62.5	0.0442	0.04245	0.89315	1.0075
31.25	0.0435	0.04465	0.52185	0.65785
15.625	0.0448	0.04455	0.26055	0.3648

Figur 1C-D

Geometric mean on live cells (B16GP75) n=3 --> first sample is depicted in histogram

concentration ug/ml

	IgG1 wt			IgG1 mut	
140	142	145	152	153	157
	IgG3 wt			IgG3 mut	
154	154	158	158	159	163
	mIgG2a			MG4	
644	681	689	32.4	15	15.8
	HEPC				
6.74	6.82	6.57			

Figure 1E

concentra	IgG1 wt			IgG1 mut			IgG3 wt		
0.001	4	3.92	3.85	3.98	4.03	3.98	4.2	4.08	4.21
0.01	7.16	7.33	7.28	10.1	9.97	9.38	8.17	7.9	7.9
0.1	23	23.4	23.7	30.1	28.3	29.2	24.3	25	24.5
1	98.4	95.3	92.2	115	112	110	105	102	99.3
10	140	142	145	152	153	157	154	154	158
100	148	148	146	160	162	164	166	168	167

IgG3 mut			HEPC		
4.09	4.52	5.42	6.14	6.11	6.03
9.53	9.75	9.66	4.75	4.87	4.76
30.7	30.9	31.2	4.75	4.81	4.57
117	116	114	4.69	4.68	4.66
158	159	163	6.74	6.82	6.57
168	172	174	8.19	7.91	11.2

Geometric mean of live cells, n=3

Figure 1F

concentra	mIgG2a			MG4		
0.001	5.72	6.09	6.12	5.64	5.68	5.44
0.01	25.6	25	25.2	4.48	4.83	4.89
0.1	100	105	109	4.72	4.68	4.55
1	420	405	408	8.88	4.5	4.49
10	644	681	689	32.4	15	15.8
100	696	712	722	8.08	5.07	6.18

Geometric mean (live cells) n=3

Raw data for figure 2

			excl debris | Freq. of Parent	excl debris/B10F10 | Freq. of Parent	excl debris/double | Freq. of Parent
20111021 MB fago B16.001	1 µg/ml	IgG1 wt	82,4	8,84	6,11
20111021 MB fago B16.002	1 µg/ml	IgG1 wt	84,5	9,63	4,4
20111021 MB fago B16.003	1 µg/ml	IgG1 wt	85,7	11,2	6,53
20111021 MB fago B16.004	1 µg/ml	IgG1 H435R	84,5	9,37	6,66
20111021 MB fago B16.005	1 µg/ml	IgG1 H435R	85	9,28	6,31
20111021 MB fago B16.006	1 µg/ml	IgG1 H435R	85,2	10,5	6,57
20111021 MB fago B16.007	1 µg/ml	IgG3 wt	87,1	17,8	5,05
20111021 MB fago B16.008	1 µg/ml	IgG3 wt	86,4	18,5	4,87
20111021 MB fago B16.009	1 µg/ml	IgG3 wt	86,2	18,4	5,62
20111021 MB fago B16.010	1 µg/ml	IgG3 R435H	86,7	15,2	4,71
20111021 MB fago B16.011	1 µg/ml	IgG3 R435H	87,9	19,9	4
20111021 MB fago B16.012	1 µg/ml	IgG3 R435H	87	17,1	3,9
20111021 MB fago B16.013	1 µg/ml	mIgG2a	86,8	7,35	7,92
20111021 MB fago B16.014	1 µg/ml	mIgG2a	87,1	9,22	6,61
20111021 MB fago B16.015	1 µg/ml	mIgG2a	87,3	8,33	7,09
20111021 MB fago B16.016	1 µg/ml	MG4	87	17,6	3,42
20111021 MB fago B16.017	1 µg/ml	MG4	88,6	22,4	4
20111021 MB fago B16.018	1 µg/ml	MG4	87,9	20,5	3,5
20111021 MB fago B16.019	1 µg/ml	HEPC	86,8	20,8	3,33
20111021 MB fago B16.020	1 µg/ml	HEPC	87,6	20,9	3,44
20111021 MB fago B16.021	1 µg/ml	HEPC	88,2	21,3	3,26
20111021 MB fago B16.022	1 µg/ml	no a.b.	89	21,7	3,08
20111021 MB fago B16.023	1 µg/ml	no a.b.	85,4	17,4	2,96
20111021 MB fago B16.024	0,1 µg/ml	IgG1 wt	87,2	16	3,97
20111021 MB fago B16.025	0,1 µg/ml	IgG1 wt	87,3	18,1	4,14
20111021 MB fago B16.026	0,1 µg/ml	IgG1 wt	88,5	18,2	4,98
20111021 MB fago B16.027	0,1 µg/ml	IgG1 H435R	87,7	15,3	4,17
20111021 MB fago B16.028	0,1 µg/ml	IgG1 H435R	88,9	16,4	4,34
20111021 MB fago B16.029	0,1 µg/ml	IgG1 H435R	86,7	16,4	4,14
20111021 MB fago B16.030	0,1 µg/ml	IgG3 wt	88,6	18,8	3,41
20111021 MB fago B16.031	0,1 µg/ml	IgG3 wt	89,6	17,2	2,65
20111021 MB fago B16.032	0,1 µg/ml	IgG3 wt	88,1	20,2	4,55
20111021 MB fago B16.033	0,1 µg/ml	IgG3 R435H	88	15,8	3,63
20111021 MB fago B16.034	0,1 µg/ml	IgG3 R435H	87,6	17,4	3,33
20111021 MB fago B16.035	0,1 µg/ml	IgG3 R435H	88,4	17,2	3,12
20111021 MB fago B16.036	0,1 µg/ml	mIgG2a	87,5	15,3	3,4
20111021 MB fago B16.037	0,1 µg/ml	mIgG2a	87,9	18,6	3,08
20111021 MB fago B16.038	0,1 µg/ml	mIgG2a	89,1	18,6	4,39
20111021 MB fago B16.039	0,1 µg/ml	MG4	89,1	19,1	3,15
20111021 MB fago B16.040	0,1 µg/ml	MG4	89,3	20,1	1,62
20111021 MB fago B16.041	0,1 µg/ml	MG4	88,9	19,8	2,74
20111021 MB fago B16.042	0,1 µg/ml	HEPC	87,7	21,3	2,86
20111021 MB fago B16.043	0,1 µg/ml	HEPC	88,3	20,6	2,09
20111021 MB fago B16.044	0,1 µg/ml	HEPC	88,4	20	1,91
20111021 MB fago B16.045	0,1 µg/ml	no a.b.	87	19,2	2,27
20111021 MB fago B16.046	0,1 µg/ml	no a.b.	86,9	18,3	1,8
20111021 MB fago B16.047	0,1 µg/ml	no a.b.	88,2	20	2,43
20111021 MB fago B16.048	0,01 µg/ml	IgG1 wt	87,3	22,6	1,79
20111021 MB fago B16.049	0,01 µg/ml	IgG1 wt	87,7	22,6	1,7
20111021 MB fago B16.050	0,01 µg/ml	IgG1 wt	86,5	21,2	1,91
20111021 MB fago B16.051	0,01 µg/ml	IgG1 H435R	86,7	16,6	1,56
20111021 MB fago B16.052	0,01 µg/ml	IgG1 H435R	87,6	22,6	2,94
20111021 MB fago B16.053	0,01 µg/ml	IgG1 H435R	88,8	20,8	1,9
20111021 MB fago B16.054	0,01 µg/ml	IgG3 wt	87,4	20,4	1,64
20111021 MB fago B16.055	0,01 µg/ml	IgG3 wt	88	21	2,57
20111021 MB fago B16.056	0,01 µg/ml	IgG3 wt	87,2	24,5	2,42
20111021 MB fago B16.057	0,01 µg/ml	IgG3 R435H	85,1	17,5	2,39
20111021 MB fago B16.058	0,01 µg/ml	IgG3 R435H	85,5	18,8	2,31
20111021 MB fago B16.059	0,01 µg/ml	IgG3 R435H	84,5	15,5	1,59
20111021 MB fago B16.060	0,01 µg/ml	mIgG2a	86,1	18,4	1,98
20111021 MB fago B16.061	0,01 µg/ml	mIgG2a	86,3	16,8	1,9
20111021 MB fago B16.062	0,01 µg/ml	mIgG2a	87,2	21,3	2
20111021 MB fago B16.063	0,01 µg/ml	MG4	85,4	17,4	2,44
20111021 MB fago B16.064	0,01 µg/ml	MG4	85,9	19,6	17,2
20111021 MB fago B16.065	0,01 µg/ml	MG4	87,6	21,7	3,11
20111021 MB fago B16.066	0,01 µg/ml	HEPC	85,9	21,2	2,62
20111021 MB fago B16.067	0,01 µg/ml	HEPC	85,8	19	4,18
20111021 MB fago B16.068	0,01 µg/ml	HEPC	84,8	18,9	2,3
20111021 MB fago B16.069	0,01 µg/ml	no a.b.	85,4	15,9	2,03
20111021 MB fago B16.070	0,01 µg/ml	no a.b.	86,2	21,1	3,88
20111021 MB fago B16.071	0,01 µg/ml	no a.b.	85,8	17,5	3,94
20111021 MB fago B16.072			88,2	11,6	0,038
20111021 MB fago B16.073			67,8	1,25	0,29
20111021 MB fago B16.074			96,6	1,25	0
20111021 MB fago B16.075			96,7	99,6	0,012
Mean			87,1	18,4	3,52
SD			3,06	10,6	2,31

B16	1ug						HepC=100%
IgG1 wt	8,84	9,63	11,2	42,09524	45,85714	53,33333	
IgG1 mut	9,37	9,28	10,5	44,61905	44,19048	50	
IgG3 wt	17,8	18,5	18,4	84,7619	88,09524	87,61905	
IgG3 mut	15,2	19,9	17,1	72,38095	94,7619	81,42857	
mIgG2a	7,35	9,22	8,33	35	43,90476	39,66667	
HEPC	20,8	20,9	21,3	99,04762	99,52381	101,4286	**21**
MG4	17,6	22,4	20,5	83,80952	106,6667	97,61905	
no mAb	21,7	17,4		103,3333	82,85714	0	

double	average	double							HepC=1
IgG1 wt	5,68	IgG1 wt	6,11	4,4	6,53	1,827517	1,316052	1,953141	
IgG1 mut	6,513333	IgG1 mut	6,66	6,31	6,57	1,992024	1,887338	1,965105	
IgG3 wt	5,18	IgG3 wt	5,05	4,87	5,62	1,510469	1,45663	1,680957	
IgG3 mut	4,203333	IgG3 mut	4,71	4	3,9	1,408774	1,196411	1,1665	
mIgG2a	7,206667	mIgG2a	7,92	6,61	7,09	2,368893	1,977069	2,120638	
HEPC	3,343333	HEPC	3,33	3,44	3,26	0,996012	1,028913	0,975075	**3,343333**
MG4	3,64	MG4	3,42	4	3,5	1,022931	1,196411	1,046859	
no mAb	3,02	no mAb	3,08	2,96		0,921236	0,885344	0	

B16	0,1ug						HepC=100%
IgG1 wt	16	18,1	18,2	77,54443	87,72213	88,20679	
IgG1 mut	15,3	16,4	16,4	74,15186	79,48304	79,48304	
IgG3 wt	18,8	17,2	20,2	91,1147	83,36026	97,89984	
IgG3 mut	15,8	17,4	17,2	76,57512	84,32956	83,36026	
mIgG2a	15,3	18,6	18,6	74,15186	90,1454	90,1454	
HEPC	21,3	20,6	20	103,231	99,83845	96,93053	**20,63333**
MG4	19,1	20,1	19,8	92,56866	97,41519	95,96123	
no mAb	19,2	18,3	20	93,05331	88,69144	96,93053	

double	average	double							HepC=1
IgG1 wt	4,363333	IgG1 wt	3,97	4,14	4,98	1,736152	1,810496	2,177843	
IgG1 mut	4,216667	IgG1 mut	4,17	4,34	4,14	1,823615	1,897959	1,810496	
IgG3 wt	3,536667	IgG3 wt	3,41	2,65	4,55	1,491254	1,158892	1,989796	
IgG3 mut	3,36	IgG3 mut	3,63	3,33	3,12	1,587464	1,456268	1,364431	
mIgG2a	3,623333	mIgG2a	3,4	3,08	4,39	1,48688	1,346939	1,919825	
HEPC	2,286667	HEPC	2,86	2,09	1,91	1,250729	0,913994	0,835277	**2,286667**
MG4	2,503333	MG4	3,15	1,62	2,74	1,377551	0,708455	1,198251	
no mAb	2,166667	no mAb	2,27	1,8	2,43	0,992711	0,787172	1,062682	

B16	0,01						HepC=100%
IgG1 wt	22,6	22,6	21,2	114,7208	114,7208	107,6142	
IgG1 mut	16,6	22,6	20,8	84,26396	114,7208	105,5838	
IgG3 wt	20,4	21	24,5	103,5533	106,599	124,3655	
IgG3 mut	17,5	18,8	15,5	88,83249	95,43147	78,6802	
mIgG2a	18,4	16,8	21,3	93,40102	85,27919	108,1218	
HEPC	21,2	19	18,9	107,6142	96,4467	95,93909	**19,7**
MG4	17,4	19,6	21,7	88,32487	99,49239	110,1523	
no mAb	15,9	21,1	17,5	80,71066	107,1066	88,83249	

double	average	double							HepC=1
IgG1 wt	1,8	IgG1 wt	1,79	1,7	1,91	0,727642	0,691057	0,776423	
IgG1 mut	2,133333	IgG1 mut	1,56	2,94	1,9	0,634146	1,195122	0,772358	
IgG3 wt	2,21	IgG3 wt	1,64	2,57	2,42	0,666667	1,044715	0,98374	
IgG3 mut	2,096667	IgG3 mut	2,39	2,31	1,59	0,971545	0,939024	0,646341	
mIgG2a	1,96	mIgG2a	1,98	1,9	2	0,804878	0,772358	0,813008	
HEPC	3,033333	HEPC	2,62	4,18	2,3	1,065041	1,699187	0,934959	**2,46**
MG4	2,775	MG4	2,44	17,2	3,11	0,99187	6,99187	1,264228	
no mAb	3,283333	no mAb	2,03	3,88	3,94	0,825203	1,577236	1,601626	

Raw data for figure 3

MCB12-26 exp 8

50µg/mouse	*mouse #*	Total tumour count			Total tumour area			1<br><1mm	1<br>1mm	2<br>2mm	3<br>3mm	4<br>4mm	5<br>5mm	6<br>6mm	7<br>7mm	8<br>8mm	9<br>9mm	10<br>10mm
IgG1 wt	*1*	0	4,6	average	0	5,8	average											
	2	0	2,636285	sem	0													
	3	12			15			4	6	1	1							
Group 1	*4*	8			11				5	3								
	5	3			3				3									
IgG1 H435R	*6*	0	6,6	average	0	12,8	average											
	7	9	1,890767	sem	23					6	2		1					
Group 2	*8*	9			16				2	7								
	9	8			16					8								
	10	7			9				6		1							
IgG3 wt	*11*	5	31,33333	average	20	50,5	average		1		3							1
	12	6	13,3741	sem	20					4				2				
Group 3	*13*	33			47			8	14	8	3							
	14	42			66				28	10	1		3					
	15	77			106				52	23		2						
	16	25			44				18	5				1				1
IgG3 R435H	*17*	57	40,16667	average	77	53,16667	average	25	16	15				1				
	18	85	13,28313	sem	91			18	62	4	1							
Group 4	*19*	33			62				19	10			1	3				
	20	33			47				21	10	2							
	21	14			23				5	9								
	22	19			19			4	15									
IgG2A	*23*	0	0,666667	average	0	0,666667	average	0										
	24	0	0,57735	sem	0			0										
Group 5	*25*	2			2				2									
Isotype	*26*	126	87	average	172	143,2	average	48	55		23							
	27	106	17,6706	sem	209				39	33	32	2						
Groupe 6	*28*	32			53				19	12								1
	29	92			171				15	75	2							
	30	79			111			35	23	10	11							

Raw data for sup figure 2A

		With PMN					Without PMN				
	mAb conc (ug/ml)	no mAb	2	1	0,1	0,01	no mAb	2	1	0,1	0,01
	no mAb	42797,33					34893,33				
		39788,33					33928,33				
		39266,33					34388,33				
	IgG1 TNP		49222,33	38162,33	40414,33	38953,33		35852,33	31951,33	32980,33	35607,33
			42329,33	41810,33	36105,33	39250,33		35238,33	35180,33	32843,33	34797,33
			42811,33	36849,33	33626,33	36599,33		38301,33	35649,33	33113,33	33587,33
	IgG3 TNP		37654,33	36424,33	36791,33	34803,33		36629,33	34231,33	33813,33	35149,33
			40276,33	38982,33	39327,33	37428,33		36993,33	35710,33	35787,33	35080,33
			42476,33	36603,33	37961,33	37434,33		38921,33	37486,33	39311,33	38846,33
	Serum IgA		33561,33	34457,33	36453,33	35372,33		34158,33	33724,33	32989,33	34005,33
			35437,33	36797,33	34285,33	41166,33		33347,33	33490,33	33253,33	32960,33
			36563,33	39457,33	41145,33	36802,33		36919,33	38084,33	37381,33	37382,33
	IgA TA99		36018,33	38943,33	44727,33	44723,33		36168,33	34922,33	33819,33	33985,33
			33544,33	35826,33	39870,33	36245,33		33336,33	33982,33	33722,33	32903,33
Plate 2/4			29972,33	32648,33	36225,33	35935,33		33283,33	32458,33	34343,33	35972,33
	no mAb	38071,33					32374,67				
		31617,33					32024,67				
		32758,33					31400,67				
	IgG1 wt		28746,33	27745,33	29178,33	29441,33		35772,67	33030,67	33516,67	32168,67
			35815,33	33617,33	32623,33	32055,33		34187,67	31710,67	31906,67	32446,67
			39773,33	34636,33	34896,33	33457,33		34782,67	30788,67	30112,67	29150,67
	IgG1 H435R		36484,33	35006,33	35512,33	37226,33		33367,67	30479,67	29847,67	30154,67
			37396,33	38309,33	33864,33	33759,33		33800,67	31029,67	30832,67	31154,67
			36584,33	36353,33	35882,33	35587,33		34120,67	34339,67	33827,67	35530,67
	IgG3 wt		35305,33	34577,33	34569,33	32410,33		28870,67	30445,67	29903,67	30818,67
			34434,33	33252,33	32198,33	33412,33		31147,67	30026,67	30617,67	30020,67
			34688,33	37914,33	34533,33	34603,33		33296,67	33166,67	33834,67	33711,67
	IgG3 R435H		30731,33	35479,33	38266,33	36210,33		30896,67	32198,67	32072,67	31802,67
			33688,33	30473,33	32179,33	31783,33		30905,67	29912,67	29264,67	30967,67
Plate 1/3			32603,33	32134,33	30147,33	31392,33		28495,67	28961,67	30118,67	30461,67

no mAb Average		
	plate 1	34149,00
	plate 2	40617,33
	plate 3	31933,33
	plate 4	34403,33

Relative to no mAb

		With PMN					Without PMN				
	mAb conc (ug/ml)	no mAb	2	1	0,1	0,01	no mAb	2	1	0,1	0,01
	no mAb	105,37					101,42				
		97,96					98,62				
		96,67					99,96				
	IgG1 TNP		121,19	93,96	99,50	95,90		104,21	92,87	95,86	103,50
			104,21	102,94	88,89	96,63		102,43	102,26	95,47	101,15
			105,40	90,72	82,79	90,11		111,33	103,62	96,25	97,63
	IgG3 TNP		92,71	89,68	90,58	85,69		106,47	99,50	98,29	102,17
			99,16	95,97	96,82	92,15		107,53	103,80	104,02	101,97
			104,58	90,12	93,46	92,16		113,13	108,96	114,27	112,91
	Serum IgA		82,63	84,83	89,75	87,09		99,29	98,03	95,89	98,84
			87,25	90,60	84,41	101,35		96,93	97,35	96,66	95,81
			90,02	97,14	101,30	90,61		107,31	110,70	108,66	108,66
	IgA TA99		88,68	95,88	110,12	110,11		105,13	101,51	98,30	98,79
			82,59	88,20	98,16	89,24		96,90	98,78	98,02	95,64
Plate 2/4			73,79	80,38	89,19	88,47		96,74	94,35	99,83	104,56
	no mAb	111,49					101,38				
		92,59					100,29				
		95,93					98,33				
	IgG1 wt		84,18	81,25	85,44	86,21		112,02	103,44	104,96	100,74
			104,88	98,44	95,53	93,87		107,06	99,30	99,92	101,61
			116,47	101,43	102,19	97,97		108,92	96,42	94,30	91,29
	IgG1 H435R		106,84	102,51	103,99	109,01		104,49	95,45	93,47	94,43
			109,51	112,18	99,17	98,86		105,85	97,17	96,55	97,56
			107,13	106,46	105,08	104,21		106,85	107,54	105,93	111,27
	IgG3 wt		103,39	101,25	101,23	94,91		90,41	95,34	93,64	96,51
			100,84	97,37	94,29	97,84		97,54	94,03	95,88	94,01
			101,58	111,03	101,13	101,33		104,27	103,86	105,95	105,57
	IgG3 R435H		89,99	103,90	112,06	106,04		96,75	100,83	100,44	99,59
			98,65	89,24	94,23	93,07		96,78	93,67	91,64	96,98
Plate 1/3			95,47	94,10	88,28	91,93		89,23	90,69	94,32	95,39

2ug/ml

With PMN	no mAb	Serum IgA	IgA TA99	IgG1 TNP	IgG3 TNP	IgG1 wt	IgG1 H435F	IgG3 wt	IgG3 R435H
	105,37	82,63	88,68	121,19	92,71				
	97,96	87,25	82,59	104,21	99,16				
	96,67	90,02	73,79	105,40	104,58				
	111,49					84,18	106,84	103,39	89,99
	92,59					104,88	109,51	100,84	98,65
	95,93					116,47	107,13	101,58	95,47

1ug/ml

With PMN	no mAb	Serum IgA	IgA TA99	IgG1 TNP	IgG3 TNP	IgG1 wt	IgG1 H435F	IgG3 wt	IgG3 R435H
		84,83	95,88	93,96	89,68				
		90,60	88,20	102,94	95,97				
		97,14	80,38	90,72	90,12				
						81,25	102,51	101,25	103,90
						98,44	112,18	97,37	89,24
						101,43	106,46	111,03	94,10

0,1ug/ml

With PMN	no mAb	Serum IgA	IgA TA99	IgG1 TNP	IgG3 TNP	IgG1 wt	IgG1 H435F	IgG3 wt	IgG3 R435H
		89,75	110,12	99,50	90,58				
		84,41	98,16	88,89	96,82				
		101,30	89,19	82,79	93,46				
						85,44	103,99	101,23	112,06
						95,53	99,17	94,29	94,23
						102,19	105,08	101,13	88,28

0,01ug/ml

With PMN	no mAb	Serum IgA	IgA TA99	IgG1 TNP	IgG3 TNP	IgG1 wt	IgG1 H435F	IgG3 wt	IgG3 R435H
		87,09	110,11	95,90	85,69				
		101,35	89,24	96,63	92,15				
		90,61	88,47	90,11	92,16				
						86,21	109,01	94,91	106,04
						93,87	98,86	97,84	93,07
						97,97	104,21	101,33	91,93

2ug/ml

Without PMN	no mAb	Serum IgA	IgA TA99	IgG1 TNP	IgG3 TNP	IgG1 wt	IgG1 H435F	IgG3 wt	IgG3 R435H
	101,42	99,29	105,13	104,21	106,47				
	98,62	96,93	96,90	102,43	107,53				
	99,96	107,31	96,74	111,33	113,13				
	101,38					112,02	104,49	90,41	96,75
	100,29					107,06	105,85	97,54	96,78
	98,33					108,92	106,85	104,27	89,23

1ug/ml

Without PMN	no mAb	Serum IgA	IgA TA99	IgG1 TNP	IgG3 TNP	IgG1 wt	IgG1 H435F	IgG3 wt	IgG3 R435H
		98,03	101,51	92,87	99,50				
		97,35	98,78	102,26	103,80				
		110,70	94,35	103,62	108,96				
						103,44	95,45	95,34	100,83
						99,30	97,17	94,03	93,67
						96,42	107,54	103,86	90,69

0,1ug/ml

Without PMN	no mAb	Serum IgA	IgA TA99	IgG1 TNP	IgG3 TNP	IgG1 wt	IgG1 H435F	IgG3 wt	IgG3 R435H
		95,89	98,30	95,86	98,29				
		96,66	98,02	95,47	104,02				
		108,66	99,83	96,25	114,27				
						104,96	93,47	93,64	100,44
						99,92	96,55	95,88	91,64
						94,30	105,93	105,95	94,32

0,01ug/ml

Without PMN	no mAb	Serum IgA	IgA TA99	IgG1 TNP	IgG3 TNP	IgG1 wt	IgG1 H435F	IgG3 wt	IgG3 R435H
		98,84	98,79	103,50	102,17				
		95,81	95,64	101,15	101,97				
		108,66	104,56	97,63	112,91				
						100,74	94,43	96,51	99,59
						101,61	97,56	94,01	96,98
						91,29	111,27	105,57	95,39

Raw data for sup figure 2B

ADCC+ plate 1 berekening killing CTB

	IgG1 wt			IgG1 H435R			TNP IgG1		
	2	3	4	5	6	7	8	9	10
B	588,627	449,787	545,742	409,578	651,066	956,094	284,802	337,566	340,368
C	664,554	638,103	711,72	551,523	543,837	549,585	254,298	314,457	308,571
D	696,27	744,864	633,057	465,507	395,421	421,944	241,338	306,915	356,838
E	718,101	789,27	604,299	459,693	491,568	445,641	266,601	325,755	347,994
F	442,911	493,173	648,432	402,411	501,333	595,251	311,076	347,994	367,02

ADCC+ plate 2 berekening killing CTB

	IgG3 wt			IgG3 R435H			TNP IgG3		
B	698,502	2650,011	4181,211	1124,229	1423,827	2018,073	547,296	980,316	733,32
C	628,47	1413,627	1533,648	939,21	905,58	1110,87	985,668	1924,698	517,284
D	1030,359	1687,521	856,662	657,855	771,021	774,603	815,886	720,342	557,883
E	900,942	919,74	608,361	470,712	717,156	549,39	811,986	570,999	613,293
F	534,987	789,213	573,228	566,574	891,783	403,218	1110,87	652,356	484,368

ADCC+		iso hIgG1	iso hIgG3	TA99 hIgG1 wt	TA99 hIgG1 H435R	TA99 hIgG3 wt	TA99 hIgG3 R435H
	0	284,802	547,296	588,627	409,578	698,502	1124,229
		337,566	980,316	449,787	651,066	2650,011	1423,827
		340,368	733,32	545,742	956,094	4181,211	2018,073
	0,01	254,298	985,668	664,554	551,523	628,47	939,21
		314,457	1924,698	638,103	543,837	1413,627	905,58
		308,571	517,284	711,72	549,585	1533,648	1110,87
	0,1	241,338	815,886	696,27	465,507	1030,359	657,855
		306,915	720,342	744,864	395,421	1687,521	771,021
		356,838	557,883	633,057	421,944	856,662	774,603
	1	266,601	811,986	718,101	459,693	900,942	470,712
		325,755	570,999	789,27	491,568	919,74	717,156
		347,994	613,293	604,299	445,641	608,361	549,39
	10	311,076	1110,87	442,911	402,411	534,987	566,574
		347,994	652,356	493,173	501,333	789,213	891,783
		367,02	484,368	648,432	595,251	573,228	403,218

?

plate 1		plate 2	
Well	conc in sup	Well	conc in sup
B2	588,627	B2	698,502
B3	449,787	B3	2650,011
B4	545,742	B4	4181,211
C2	664,554	C2	628,47
C3	638,103	C3	1413,627
C4	711,72	C4	1533,648
D2	696,27	D2	1030,359
D3	744,864	D3	1687,521
D4	633,057	D4	856,662
E2	718,101	E2	900,942
E3	789,27	E3	919,74
E4	604,299	E4	608,361
F2	442,911	F2	534,987
F3	493,173	F3	789,213
F4	648,432	F4	573,228
B5	409,578	B5	1124,229
B6	651,066	B6	1423,827
B7	956,094	B7	2018,073
C5	551,523	C5	939,21
C6	543,837	C6	905,58
C7	549,585	C7	1110,87
D5	465,507	D5	657,855
D6	395,421	D6	771,021
D7	421,944	D7	774,603
E5	459,693	E5	470,712
E6	491,568	E6	717,156
E7	445,641	E7	549,39
F5	402,411	F5	566,574
F6	501,333	F6	891,783
F7	595,251	F7	403,218
B8	284,802	B8	547,296
B9	337,566	B9	980,316
B10	340,368	B10	733,32
C8	254,298	C8	985,668
C9	314,457	C9	1924,698
C10	308,571	C10	517,284
D8	241,338	D8	815,886
D9	306,915	D9	720,342
D10	356,838	D10	557,883
E8	266,601	E8	811,986
E9	325,755	E9	570,999
E10	347,994	E10	613,293
F8	311,076	F8	1110,87
F9	347,994	F9	652,356
F10	367,02	F10	484,368
B11	232,074	B11	#VALUE!
C11	196,35	C11	#VALUE!
D11	212,934	D11	#VALUE!
B12	364,953	B12	589,227
C12	364,953	C12	502,263
D12	389,73	D12	414,183
